# Navigating the Diagnostic and Management Challenges of Suspected Cardiac Amyloidosis in Resource‐Limited Settings: Ethiopia Experience

**DOI:** 10.1002/ccr3.9582

**Published:** 2024-11-18

**Authors:** Sura Markos, Yegzeru Belete, Betre Bikamo, Demelash Ataro

**Affiliations:** ^1^ Division of Cardiology, Department of Internal Medicine, College of Medicine and Health Sciences Hawassa University Hawassa Ethiopia; ^2^ School of Medicine College of Medicine and Health Sciences, Hawassa University Hawassa Ethiopia; ^3^ Department of Internal Medicine, College of Medicine and Health Sciences Hawassa University Hawassa Ethiopia; ^4^ Department of Emergency Medicine and Critical Care, College of Medicine and Health Sciences Hawassa University Hawassa Ethiopia

**Keywords:** amyloidosis, cardiac amyloidosis, Ethiopia, resource limited, restrictive cardiomyopathy

## Abstract

This report discusses the diagnostic and therapeutic complexities encountered in resource‐limited environments when assessing patients suspected of cardiac amyloidosis (CA). The study describes a case involving a patient who initially presented with heart failure symptoms and eventually received a CA diagnosis after 13 months and four primary physician visits, primarily based on discrepancies observed between electrocardiographic and echocardiographic findings, alongside elevated cardiac troponin levels. The case underscores the limited awareness of CA among primary healthcare providers in Ethiopia, contributing to a higher likelihood of misdiagnosis and inappropriate treatment approaches. Additionally, the report discusses the specific challenges associated with diagnosing and managing CA patients. It advocates for essential resources such as heightened clinical suspicion, proficiency in recognizing the characteristic electrocardiogram (ECG) and echocardiographic indicators of CA, and prompt referral to cardiologists.

**Abbreviations**
ATTR: amyloid transthyretin
ATTRv: variant transthyretin amyloid
ATTRwt: wild‐type transthyretin amyloid
CA: cardiac amyloidosis
CMR: cardiac magnetic resonance
ECG: electrocardiogram
IVC: inferior vena cava
IVS: interventricular septum
LV: left ventricle
LVPW: left ventricular posterior wall
RV: right ventricle,
TTR: transthyretin

**Summary**

- Cardiac amyloidosis, although thought to be rare, presents significant challenges in diagnosis and treatment in resource‐limited settings, leading to delays in diagnosis that can worsen prognosis if detected late. Therefore, maintaining a high level of suspicion and utilizing tools such as Electrocardiogram and Echocardiography for early diagnosis can help improve outcomes in settings where advanced diagnostic modalities are limited.
- In resource‐limited settings, ECG and echocardiography play a pivotal role in the diagnosis of CA; even if not confirmatory.
- A dire need for cardiology service expansion nationwide would improve the care of the early suspected diagnosis of CA.
- The diagnostic challenge of CA in third‐world setups is cumbersome. To nullify the diagnostic uncertainty, cardiac centers need to be equipped with cardiac MRI, scintigraphy, genotyping, and possibly endomyocardial biopsy to confirm the diagnosis.
- Our patient's case will be a window for primary care physicians in setups like ours to see that rare diagnosis do exist and that early appropriate referral to cardiologists plays a crucial role.
- We also believe that there should be diagnostic algorithms available for resource‐constrained countries where none of the current guidelines and diagnostic algorithms can be applied to our healthcare facilities.

## 1 Background

Amyloidosis is a localized or systemic deposition disease in which proteins with unstable tertiary structures misfold, aggregate, and form amyloid fibrils that deposit with a range of chaperone proteins in the heart, kidneys, liver, gastrointestinal tract, lungs, and soft tissues [1].

Cardiac amyloidosis is a rare and often underdiagnosed condition characterized by the deposition of dysfunctional amyloid proteins in the heart, leading to progressive heart failure. Immunoglobulin light chain (AL) amyloidosis, wild‐type transthyretin amyloidosis (ATTRwt), and hereditary/variant‐type transthyretin amyloidosis (ATTRv) are the common causes of amyloid cardiomyopathy. Due to the vast breadth of cardiac involvement, the clinical symptoms vary widely. Patients with cardiac amyloidosis may present with cardiomyopathy, congestive heart failure, coronary heart disease, valvular disease, arrhythmia, and heart block [2].

Diagnostic challenges often arise due to the nonspecific symptoms, wide range of clinical manifestations, and the need for specialized tests such as cardiac magnetic resonance (CMR) imaging, endomyocardial biopsy and scintigraphy. In resource‐limited settings, access to these diagnostic tools may be limited, making the timely diagnosis of cardiac amyloidosis even more difficult. Here, we present a suspected case of cardiac amyloidosis based on the clinical picture, cardiac biomarker, ECG, and echocardiography reports in a resource constrained cardiac unit in Sub‐Saharan Africa, Ethiopia.

### 1.1 Case Presentation

This is 54‐years‐old male patient presented with progressively worsening shortness of breath over the past month. He has been experiencing exertional dyspnea for the past year, along with easy fatigability. Recently, his symptoms worsened, now also including orthopnea, paroxysmal nocturnal dyspnea, and progressive bilateral leg swelling. Despite seeking care at various local health facilities and being prescribed unspecified medications, his condition has not improved.

Two months ago, he was diagnosed with a cardiac issue and prescribed furosemide 40 mg twice daily and enalapril 5 mg twice daily at a local hospital. However, he has not seen any improvement and experiences dizziness and increased fatigue after taking enalapril. He does not have a personal or family history of hypertension or diabetes mellitus. He denies having any syncopal attacks or other non‐cardiac symptoms such as back or neck pain, tingling sensations in the extremities, difficulty walking, joint pain, or a history of surgeries. There are no features of autonomic dysfunction, such as chronic gastrointestinal issues, erectile dysfunction, or changes in bowel habits.

During the examination, the patient's blood pressure was 100/60 mmHg, with a pulse rate of 110/min regular and full. The respiratory rate was 23/min. Physical findings showed dullness and decreased air entry in the bibasilar area, predominantly on the right side. The cardiovascular examination revealed an elevated jugular venous pressure, a quiet precordium, and a systolic murmur at the left sternal border. The liver was enlarged 6 cm below the right costal margin and tender on deep palpation, with associated shifting dullness and grade ++ bilateral pitting leg edema. No other extracardiac manifestations of amyloidosis were observed.

### 1.2 Investigations

**FIGURE 1:**
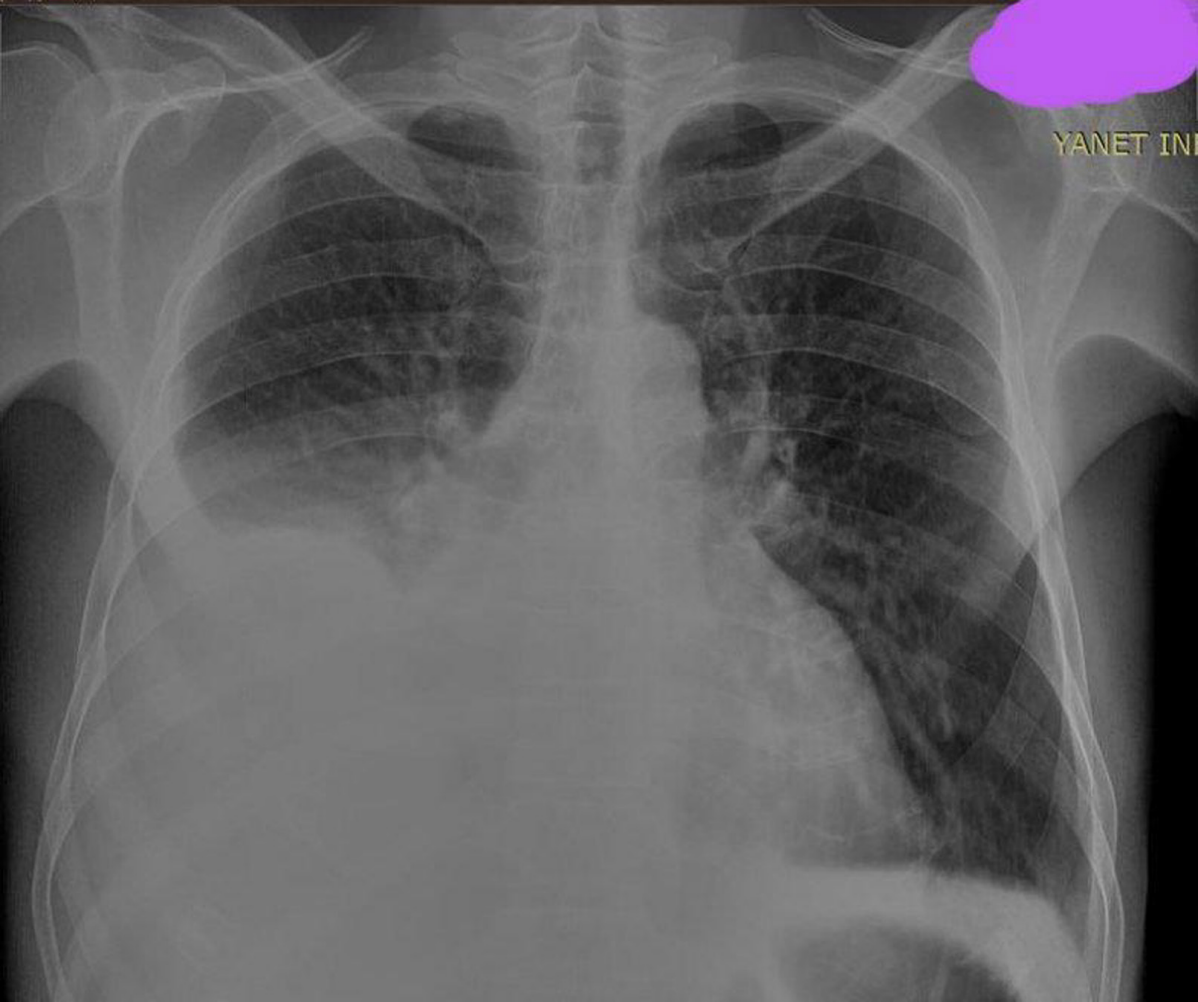
Chest X‐ray (CXR) demonstrating right side pleural effusion.

**FIGURE 2:**
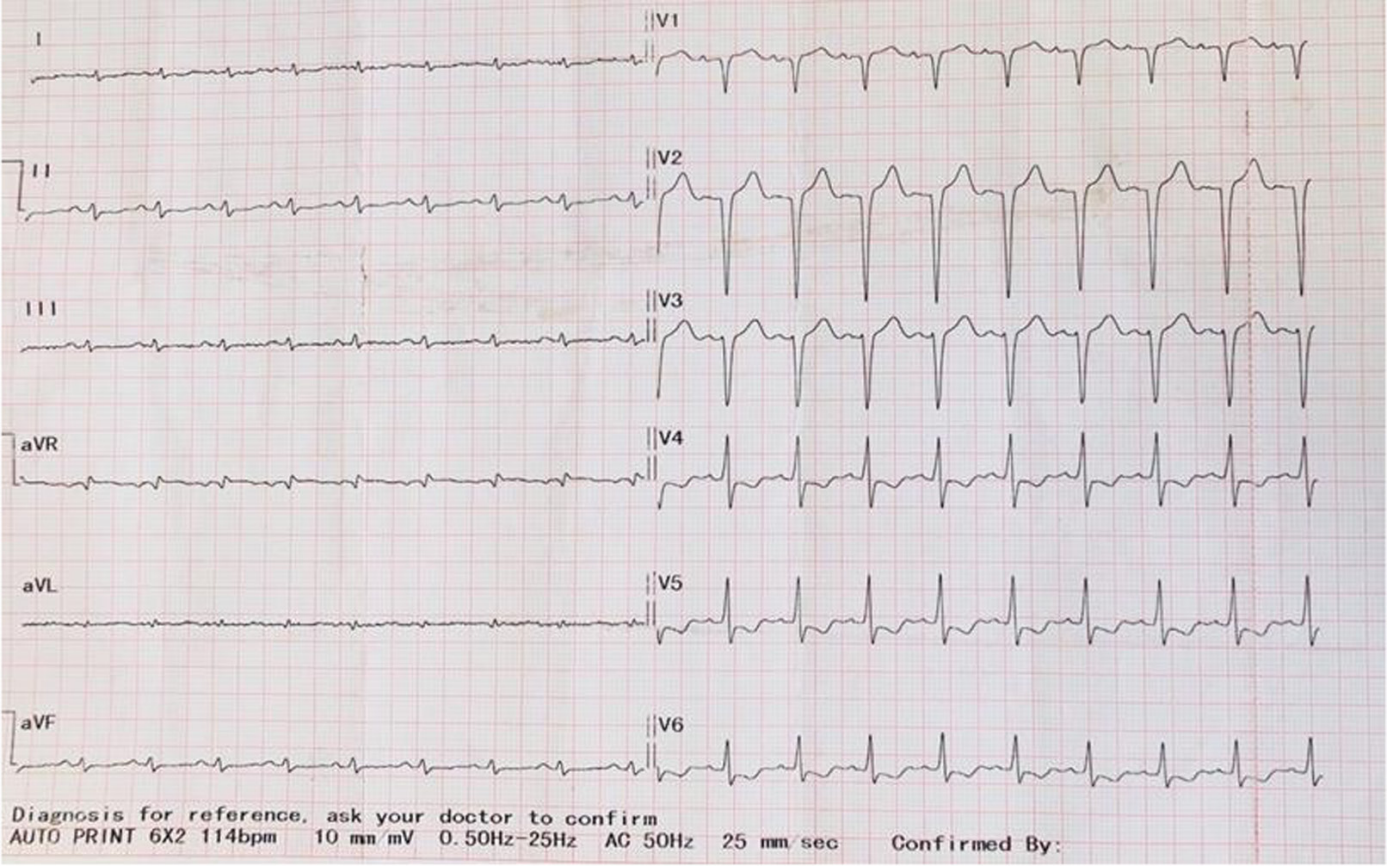
Electrocardiogram (ECG) demonstrating low voltage in the limb leads and a pseudoinfarct pattern in the V1‐3 and ST segment depression with T‐wave Inversions in V4‐6.

Laboratory results indicated normal levels of creatinine, blood urea nitrogen (BUN), serum electrolytes including magnesium and calcium, liver function tests, and normal urine analysis. The complete blood count showed a hemoglobin level of 11.7 g/dL with a mean corpuscular volume (MCV) of 91 and a mean concentration of hemoglobin (MCH) of 29. Serum troponin levels were persistently elevated on two occasions (656 and 578 ng/L: normal range in our lab ref.: < 100 ng/L). Imaging studies revealed passive hepatic congestion with hepatomegaly and also ascites and bilateral pleural effusion on abdominal ultrasound, right pleural effusion on chest X‐ray (Figure 1), and ECG showed low voltage limb leads with pseudo infarction pattern in precordial leads (Figure 2).

**FIGURE 3:**
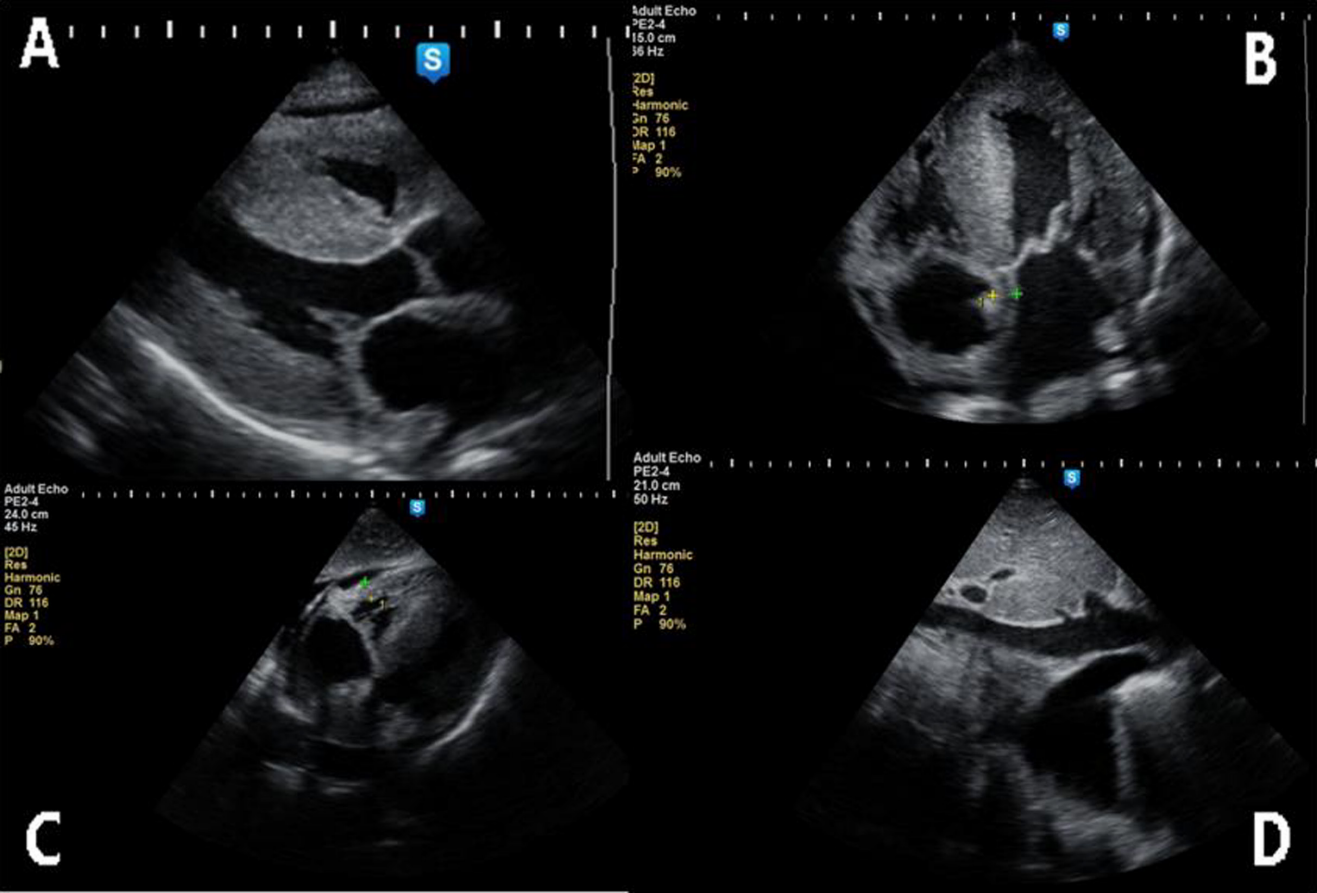
Transthoracic echocardiography (TTE): parasternal long axis showing severe left ventricular hypertrophy with small anterior pericardial effusion (A), marked septal hypertrophy, biatrial enlargement and interatrial septal hypertrophy with the granular sparkling of the myocardium on apical four‐chamber view (B), right ventricular hypertrophy with small pericardial effusion in subcostal view (C), dilated inferior vena cava (IVC) with right side pleural effusion (D).

Echocardiography showed (Figure 3 and Video 1) bi‐atrial enlargement with bi‐ventricular hypertrophy (LV: IVS: 22 mm, LVPW: 21, RV wall; 14) with interatrial septal thickening. Normal biventricular internal dimension with the granular sparkling of the myocardium. Systolic left ventricular function is normal, with an ejection fraction of 65%, and no regional wall motion abnormality was noted. The mitral inflow indices and TDI indicate the presence of marked impaired LV filling with atrial arrest (E/A ratio; 11, septal e; 3 and E/e 18). Mitral, aortic and tricuspid valves are thickened with mild calcification. The IVC is plethoric and normal pericardium with minimal pericardial effusion.

**VIDEO 1:** Transthoracic echocardiography video revealing severe biventricular hypertrophy with bi‐atrial enlargement, mitral and tricuspid valve thickening, interatrial septal thickening with myocardial sparkling and minimal pericardial effusion. Video content can be viewed at https://onlinelibrary.wiley.com/doi/10.1002/ccr3.9582

### 1.3 Differential Diagnosis

#### 1.3.1 Hypertensive Heart Disease

One of the possible differential diagnoses in this patient is hypertensive heart disease. However, the patient's lack of a previous or current history of hypertension, even though masked hypertension is still likely, the presence of a low voltage ECG instead of ECG features of left ventricular hypertrophy (LVH) despite the degree of LVH seen on echocardiography (known as ECG‐Echo discordance), along with biventricular hypertrophy, interatrial septal thickening, and myocardial sparkling (though not specific), and persistent elevation of troponin in the absence of myocardial ischemia, suggest that hypertensive heart disease is less likely in this case.

#### 1.3.2 Hypertrophic Cardiomyopathy

Cardiac amyloidosis is frequently misdiagnosed as hypertrophic cardiomyopathy (HCM). Therefore, distinguishing between the two conditions is crucial given the variety of treatment options and differences in long‐term prognosis. While CMR imaging is now the preferred method for differentiation, it was not utilized in this particular patient due to the unavailability of imaging. However, the presence of heart failure with predominant right‐sided symptoms and assessment of the QRS amplitude on an ECG helped as an initial step in distinguishing between the two conditions. High QRS voltage is typically expected in HCM, but the lack of high voltage QRS does not definitively rule out HCM. Additionally, the presence of right ventricular hypertrophy in a patient with increased left ventricular wall thickness and thickening of the interatrial septum, along with discrepancies between the ECG and echocardiogram findings, make HCM less likely in this patient even without diagnostic imaging such as CMR.

#### 1.3.3 Cardiac Amyloidosis

Cardiac amyloidosis is the most likely diagnosis for this patient based on his presentation. The patient exhibits heart failure with predominant right‐sided symptoms and intolerance to low‐dose enalapril, with symptom improvement after discontinuing the medication. Retrospectively, this suggests cardiac amyloidosis, which is further supported by typical low voltage QRS with a pseudo infarction pattern, along with echocardiographic features such as LVH (ECG‐echocardiography discordance), biventricular hypertrophy, biatrial enlargement, interatrial septal thickening, myocardial sparkling, restrictive LV filling, and typical atrial arrest on echocardiography with persistent elevation of cardiac troponin. These features, especially when observed in a patient without systemic hypertension, strongly indicate cardiac amyloidosis. Additional tests such as CMR, scintigraphy, tissue diagnosis, and genetic testing can confirm the diagnosis and differentiate it from other conditions, but the constellation of findings strongly supports the diagnosis of cardiac amyloidosis in this patient.

### 1.4 Treatment, Follow‐Up Outcome, and Future Plan

Considering cardiac amyloidosis (CA), our goal was to maintain the patient's euvolemia and optimize perfusion by addressing volume overload while considering the patient's narrow range of tolerable circulating volume. We discontinued enalapril, which led to significant clinical improvement in symptoms such as dizziness and fatigue, in conjunction with the use of furosemide and spironolactone. During the initial month, we scheduled frequent clinic visits to adjust the dosage of diuretics (furosemide and spironolactone) to ensure the patient's optimal fluid balance. The patient showed substantial symptomatic improvement during follow‐up, without requiring readmission throughout the monitoring period. Due to the patient residing in a rural area of Ethiopia where frequent visits may be challenging, we scheduled longer appointments every 2–3 months. Additionally, we provided a written report to avoid medications that could potentially worsen symptoms, such as angiotensin‐converting inhibitors/angiotensin receptor blockers and certain calcium blockers, known to cause hypotension. Given the patient's risk of atrial arrhythmias, heart blocks, and deterioration of ejection fraction, early detection and prompt treatment were prioritized, including rate control, anticoagulation, and guideline‐directed medical therapy as tolerated in case of complications. While advanced treatments like tafamidis are not readily available in Ethiopia, our approach in resource‐limited settings primarily focuses on maintaining euvolemia, alleviating symptoms, and promptly addressing complications to improve patient outcomes.

## 2 Discussion

Early diagnosis of CA requires high clinical suspicion, knowledge of cardiac and extracardiac red flags, and appropriate diagnostic algorithms. Early detection provides both quality of life and functional advantages for CA individuals; however, nondiagnostic or delayed diagnosis leads to high morbidity and mortality, and diagnostic delays negatively affect cardiac function [3, 4, 5, 6].

Cardiac amyloidosis is an uncommon condition that is often not considered as a possible differential diagnosis in patients with heart failure. Due to its rarity and unclear clinical presentation, there can be a diagnostic delay even in the best‐equipped medical settings where gold‐standard diagnostic modalities, whether invasive or non‐invasive, are available. It is reported that patients may have different types of CA in a single patient, and they may even present with bizarre presentations like unexplained ascites, which will make the differential diagnosis to be unthinkable for such patients [2, 7].

The suspicion for the diagnosis of cardiac amyloidosis in our patient is raised due to the following reasons: he had no personal or family history of hypertension, and he had no other evidence for a hereditary cause of this degree of biventricular hypertrophy, granular sparkling, interatrial septal hypertrophy with low voltage, and pseudo infarction pattern on ECG; there is ECG and echocardiography discordance as well as elevated levels of serum troponin (656 and 578 ng/L) that favor cardiac amyloidosis among many causes of restrictive cardiomyopathy.

In the literature, specific signs of CA in echocardiography are well described. Diagnosing CA involves a mismatch between ECG findings (such as low QRS voltage or a pseudo‐infarction pattern in precordial leads; however, the lack of low QRS voltage on ECG is only present in about 30% of patients with cardiac amyloidosis, and thus its absence does not exclude the diagnosis) and transthoracic echocardiography revealing concentrically thickened ventricular walls with a restrictive pattern. Some authors have indicated that a septal thickness of 1.98 cm (ours was 2.2 cm) combined with low ECG voltages, in the absence of systemic hypertension, has a sensitivity of 72% and a specificity of 91% for diagnosing CA. In Chinese patients, the hallmark echocardiography result is symmetric left ventricular hypertrophy (LVH) and diastolic dysfunction concurrent with pericardial effusion, which aligns with our patient's findings. A recent study suggests that a high‐sensitivity Troponin T (hs‐TnT) level below 14 ng/L can reliably rule out the diagnosis of CA, implying that low levels of circulating biomarkers may be safely used to exclude CA and prevent further invasive or non‐invasive testing [5, 8, 9, 10].

Cardiac amyloidosis is often misdiagnosed, and the delayed diagnosis has significant consequences for patients. The average time from initial symptoms to diagnosis was 2 years. A substantial proportion of patients reported seeing at least five physicians before receiving a diagnosis of amyloidosis [1]. In Romania, the mean delay from the first presentation with cardiac symptoms to diagnosis was no longer than 24 months [3]. A study of patients at the UK National Amyloidosis Centre found a median diagnostic delay of 39 months [11]. Our patient had a 13‐month delay in diagnosis and had visited four physicians; this could have been prolonged if an early referral had not been made to a setting where a cardiology service was available, with appropriate case‐specific interpretation of echocardiographic and electrocardiographic findings.

The lack of particular diagnostic testing makes the CA diagnosis challenging. Although challenges remain, approaches for evaluating and diagnosing patients with suspected cardiac amyloidosis have significantly improved in recent years. Cardiac magnetic resonance imaging is pivotal in distinguishing cardiac amyloidosis from hypertensive heart disease and sarcoidosis. Nuclear SPECT, genotyping, and endomyocardial biopsy also play crucial roles in the diagnosis of cardiac amyloidosis, with endomyocardial biopsy serving as the gold standard diagnostic test boasting a sensitivity rate ranging from 87% to 98%and also considered 100% sensitive and specific if biopsy specimens are collected from multiple sites (four or more sites) [4, 12, 13, 14]. However, the biopsy of a surrogate site, such as the abdominal fat pad, offers varying sensitivity: 84% for AL‐CM, 45% for ATTRv‐CM, and 15% for ATTRwt‐CM [15]. Additionally, there is an acute need for increased awareness of CA among all healthcare providers, especially Internal medicine specialists and Cardiologists. None of the new approaches for evaluating and managing the CA is available in Ethiopia.

In settings with limited resources like Ethiopia, advanced imaging modalities such as CMR and scintigraphy may not be easily accessible. Therefore, the proposed most accessible and cost‐effective diagnostic tool is transthoracic echocardiography, supported by electrocardiography, and supplemented occasionally by serum and urine immunofixation, serum‐free light chains tests, serum troponin, and N‐Terminal ProBNP. Echocardiographic diagnostic clues include an increase in left ventricular wall thickness, as well as other features such as thickening of the atrioventricular valve, right ventricle free wall, and interatrial septum, bi‐atrial enlargement along with characteristics of a restrictive LV filling pattern, such as an increased E/e’ ratio, atrial arrest, and decreased mitral annular systolic velocity (s'). When combined with an ECG showing discordant QRS voltage relative to the degree of increased left ventricular wall thickness, these findings can provide valuable insights. Left ventricular global longitudinal strain (GLS) serves as a sensitive marker for detecting cardiac amyloidosis before overt LV dysfunction becomes clinically apparent, even in patients with mild ventricular hypertrophy [16, 17, 18]. However, access to this diagnostic tool is limited in Ethiopia due to a shortage of trained personnel in this specialized field and a lack of echocardiography machines necessary to perform these procedures effectively.

In general, the management approach to a patient with cardiac amyloidosis involves treatment of the underlying cause (chemotherapy for AL, TTR‐directed therapies for ATTR) and concurrent management of heart failure, arrhythmia, and accompanying symptoms [19].

Maintenance of euvolemia and optimizing perfusion in patients with cardiac amyloidosis can be a distinct challenge due to narrow range of tolerable circulating volume. Diuretics remain a cornerstone in managing fluid retention and congestion in CA patients, aiming to achieve euvolemia and alleviate symptoms of volume overload without causing volume depletion. This approach closely monitors the patient's clinical status and fluid balance parameters, with frequent modifications to diuretic dosages aimed at achieving and maintaining euvolemia. The strategy emphasized a tailored and responsive management approach, acknowledging the dynamic nature of fluid retention and ensuring optimal therapeutic efficacy while minimizing the risk of electrolyte disturbances and other potential adverse effects associated with diuretic therapy. The selection and dosage of these medications should be individualized, considering the specific clinical manifestations and hemodynamic profile of each patient with CA [19, 20].

Evidence‐based therapies that have proven beneficial in other causes of heart failure, including angiotensin‐converting enzyme (ACE) inhibitors, angiotensin receptor blockers (ARBs), angiotensin receptor neprilysin inhibitors (ARNIs), and beta blockers are generally poorly tolerated in patients with advanced cardiac amyloidosis owing to fixed stroke volume and impaired hemodynamic compensatory mechanisms and worsens hypotension and discontinuation may improve outcomes [15, 19, 20, 21].

Non‐dihydropyridine calcium channel blockers (CCBs) are usually not recommended for cardiac amyloidosis due to their potential to bind amyloid fibrils, exacerbating heart failure by slowing heart rate, reducing contractility, and increasing conduction block. TTR amyloid fibrils have a calcium‐related interaction with bone‐seeking radiotracers, suggesting a possible mechanism for heightened CCB binding. Dihydropyridine CCBs may assist in controlling blood pressure, especially in renal dysfunction, but may lead to lower‐extremity edema. While AL amyloid fibrils have a strong affinity for digoxin in vitro, Digoxin is recently reconsidered if used cautiously as adjunctive management in AF rate control [22, 23].

Recent studies showed that SGLT2 inhibitors improved symptoms, renal function, and reduced diuretic use in ATTR‐CM patients, lowering HF hospitalization and mortality risk [24]. In cases of cardiac amyloidosis complicated by atrial fibrillation, the use of warfarin or novel oral anticoagulants for anticoagulation is recommended, irrespective of CHA2DS2‐VASc risk scores [15, 22].

In the ATTR‐ACT study, tafamidis (TTR stabilizers) resulted in lower all‐cause mortality and reduction in cardiovascular hospitalizations, lower rate of decline in distance for the 6‐min walk test and in the Kansas City Cardiomyopathy Questionnaire (KCCQ‐OS) as compared with controls [25]. Society guidelines and expert consensus recommend tafamidis as a Class I recommendation for ATTR Amyloidosis to reduce all‐cause mortality and cardiovascular‐related hospitalizations [15, 19, 20, 21]. Due to its extremely high cost and limited cost‐effectiveness in resource‐constrained settings such as Africa, including Ethiopia, this therapy is not readily available and is rarely prescribed.

The proposed management protocol for Cardiac Amyloidosis in resource‐limited settings like Ethiopia should prioritize the management of heart failure and arrhythmias with the primary aim of enhancing overall quality of life. This can be achieved through the use of diuretic therapy such as furosemide or torsemide in combination with spironolactone, with frequent dose adjustments as necessary. Anticoagulation should be administered, regardless of individual risk scoring, if the patient is complicated by atrial fibrillation. Additionally, early consideration for permanent pacemaker implantation is warranted in cases where the patient develops high‐grade symptomatic heart block. It is worth noting that guideline‐directed medical therapy may have limited role in CA due to lower tolerance and exclusion from clinical trials, thus cautious use is advised when indicated. In cases of cardiac involvement in AL amyloidosis, treatment should mirror that of multiple myeloma with chemotherapy while simultaneously addressing heart failure symptoms with diuretics [26].

Major knowledge gaps were identified in different surveys among primary care providers in the United States and practicing cardiologists in the United Arab Emirates, Kuwait, Bahrain, Qatar, Oman, and Romania and health care providers in Egypt that range from lack of awareness among health care providers in Egypt up to a high level of perceived awareness in the middle east and gulf region. However, the disease remains underrecognized as a cause of HF, indicating further education is needed to increase clinician suspicion of CA so patients who may require additional testing to make an accurate diagnosis, can be identified [3, 27, 28, 29].

Even though there is currently no research in our country regarding the awareness, knowledge, level of expertise, and familiarity of primary healthcare providers or cardiologists in the diagnosis and management of CA, this represents a new area of interest that warrants thorough investigation. Understanding our position relative to other countries/regions will allow stakeholders and healthcare professionals to develop tailored algorithms for our setting, thereby reducing the risk of misdiagnosis among primary healthcare providers.

We can surmount these challenges by improving care‐provider awareness of the disease to increase clinical suspicion of CA in the early stages, improving early diagnostic capacity by making additional diagnostic tools such as cardiac scintigraphy and genetic testing more available and affordable and working to create novel therapeutic options for cardiac amyloidosis affordable and available in low‐resource settings.

## 3 Conclusion

The challenges in diagnosing and treating cardiac amyloidosis in resource‐limited settings underscore the importance of increasing awareness among health professionals and advancing diagnostic modalities. This case report emphasizes the significance of maintaining a high index of suspicion and utilizing tools like ECG and echocardiography in the diagnosis of cardiac amyloidosis. By improving awareness and diagnostic capabilities, we can better identify and manage this rare but critical condition in resource‐limited settings.

## Author Contributions

**Sura Markos:** conceptualization, data curation, investigation, supervision, validation, visualization, writing – original draft, writing – review and editing. **Yegzeru Belete:** visualization, writing – original draft, writing – review and editing. **Betre Bikamo:** investigation, validation, writing – review and editing. **Demelash Ataro:** supervision, writing – review and editing.

## Ethics Statement

The authors have nothing to report.

## Consent

The patient provided written informed consent for the publication of details including history, physical findings, laboratory reports, all images and clinical data. Written informed consent was obtained from the patient to publish this report in accordance with the journal's patient consent policy.

## Conflicts of Interest

The authors declare no conflicts of interest.

## Data Availability

The data that support the findings of this case report are available from the corresponding author upon reasonable request.
